# Oral Rehabilitation of an S-ECC Case with Orthodontic Intervention: 18 Months Follow-up

**DOI:** 10.5005/jp-journals-10005-1101

**Published:** 2010-04-15

**Authors:** Kayalvizhi Gurusamy, Raju OS, Thejo Krishna P, Neeraja R

**Affiliations:** 1 Assistant Professor, Department of Pedodontics and Preventive Dentistry, MR Ambedkar Dental College, Bengaluru, Karnataka, India; 2 Professor, Department of Pedodontics and Preventive Dentistry, Bapuji Dental College and Hospital, Davangere, Karnataka, India; 3 Assistant Professor, Department of Pedodontics and Preventive Dentistry, Terna Dental College, Mumbai, Maharashtra, India

**Keywords:** Severe-early childhood caries (S-ECC), Diet, Rehabilitation, Cavitated, Prevention, Children.

## Abstract

Severe-early childhood caries (S-ECC) is a specific form of rampant decay of primary teeth in infants, characterized by aggressive tooth destruction. This multifactorial disease in young infants is associated with the frequent use of sweetened fluids and fermentable carbohydrates over extended periods, poor oral hygiene as well as high level of mutans streptococci infection. The disease control and restoration of severely decayed primary teeth in children with S-ECC remains a challenge to general as well as pediatric dentists. This article portrays the oral rehabilitation of a five and half-year-old girl presenting with S-ECC with an 18 months follow-up.

## INTRODUCTION

Severe-early childhood caries (S-ECC) is a specific form of rampant decay of primary teeth in infants. It may be associated with infection, pain and premature loss of primary teeth.^1^ In children younger than 3 years of age, any sign of smooth surface caries is indicative of S-ECC according to American academy of pediatric dentistry (AAPD). From ages 3 to 5 years, one or more cavitated, missing or filled smooth surfaces in primary maxillary anterior teeth or filled score of ≥ 4 (age 3), or ≥ 5 (age 4), or ≥ 6 (age 5) surfaces constitutes S-ECC.^2^

Typically, the maxillary primary incisors hit the hardest, followed by the first primary molars. Intervention at the early stage is necessary to prevent the destruction of the crown and stop the caries from progressing.^3^ The treatment of children with S-ECC depends on the extent of the lesion, age and behavioral level of the child and the degree of cooperation of the parents.^4^ The disease control and restoration of severely decayed primary teeth in children with S-ECC are challenging procedures in pediatric dentistry.^5^ This case report describes the management of a five and half-year-old girl presenting with S-ECC with 18 months follow-up.

## CASE REPORT

A five and half-year-old girl accompanied by her mother reported to our department with the chief complaint of multiple decayed teeth. Her oral hygiene status was poor. Medical history was unremarkable. Behavioral history revealed that the child was anxious and reluctant to sit in the dental chair at her first visit. Mother was instructed to fill the 7 days diet record to assess the dietary status of her child. She was asked to fill all that the child takes during the day, between meals and the frequency of their intake. Dietary evaluation revealed that the child was on high cariogenic diet with frequent intake of in between meal, snacks and soft drinks.

**Figs 1A and B:**
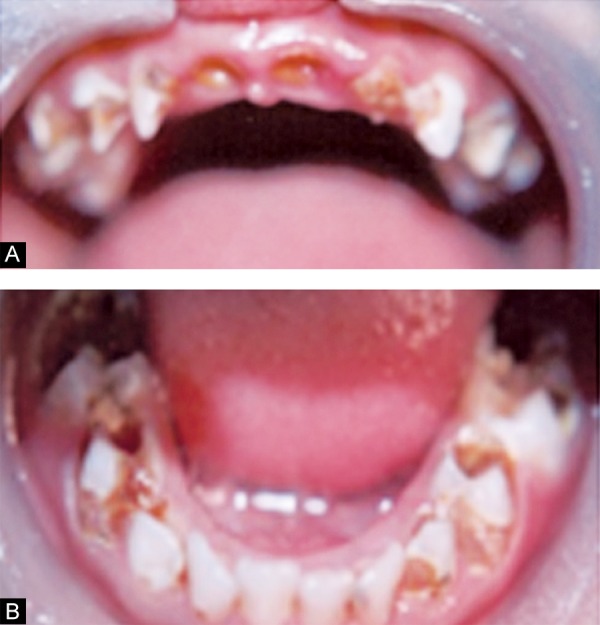
Preoperative intraoral view (A) upper arch (B) lower arch

Intraoral examination revealed multiple carious teeth involving maxillary (54, 55, 64, 65) and mandibular (74, 75, 84, 85) molars, canines (53, 63, 73, 83) and maxillary laterals (52, 62). White spot lesions and brownish discol-orations were seen in mandibular incisors (71, 72, 81, 82). Root stumps were present in relation to maxillary centrals (51, 61) (Figs 1A and B). Orthopantamogarm (OPG) confirmed the above clinical findings. Diagnosis was made based on history, clinical and radiographic findings as S-ECC.

At first visit, parents were counseled to modify their child’s diet by including the food groups from the basic diet. Reducing the frequency of cariogenic food and supplementing cariogenic food with noncariogenic items like cheese and peanut. Mother was instructed to follow oral hygiene measures. Preappointment behavior modification was performed by modeling and treatment which was initiated with the help of audiovisual aids and tell-show-do (TSD) technique.

During the second visit, dentin stabilization/ tempori-zation was done for all the molars to reduce their caries progression. Fluoride varnish application was initiated.

At third visit, all the molars were restored with miracle mix and anterior teeth with glass ionomer cement (GIC). Maxillary right first molar (54) was extracted as it could not be saved, and band and loop space maintainer was given. Pulpectomy was done in maxillary central incisors (51, 61) and sealed with GIC.

After 3 months, molars with extensive caries (74, 84, 85) restored with miracle mix were further reinforced with stainless steel crowns (SSC).

**Figs 2A and B:**
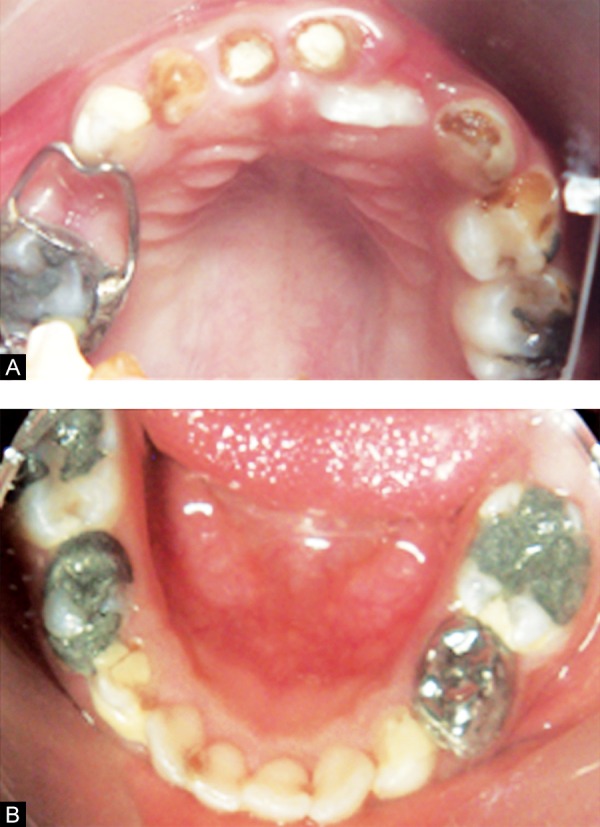
Postoperative view after 6-month follow-up (A) upper arch (B) lower arch

**Figs 3A and B:**
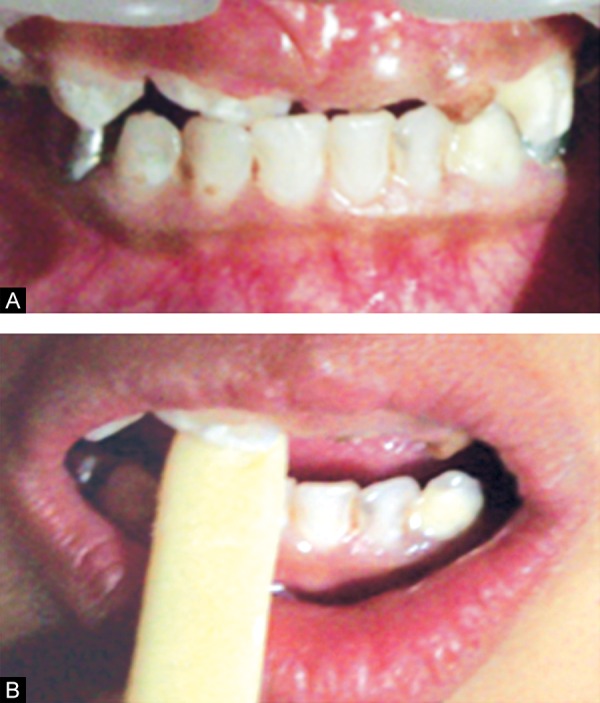
Tongue blade therapy for developing anterior crossbite correction

Six months follow-up revealed exfoliation of maxillary right central incisor (51) with the partial eruption of permanent maxillary right central incisor (11), which was in developing crossbite with the mandibular incisors (Figs 2A and B). Therefore, tongue blade therapy was planned, wherein the patient was instructed to place an ice cream stick on the palatal aspect of the tooth in crossbite. The blade was made to rest on the mandibular tooth (fulcrum) in crossbite, and the patient was asked to move the oral part of the blade upwards and forwards for 1 to 2 hours for about 2 weeks. Second round of fluoride varnish applications were done (Figs 3A and B).

**Figs 4A and B:**
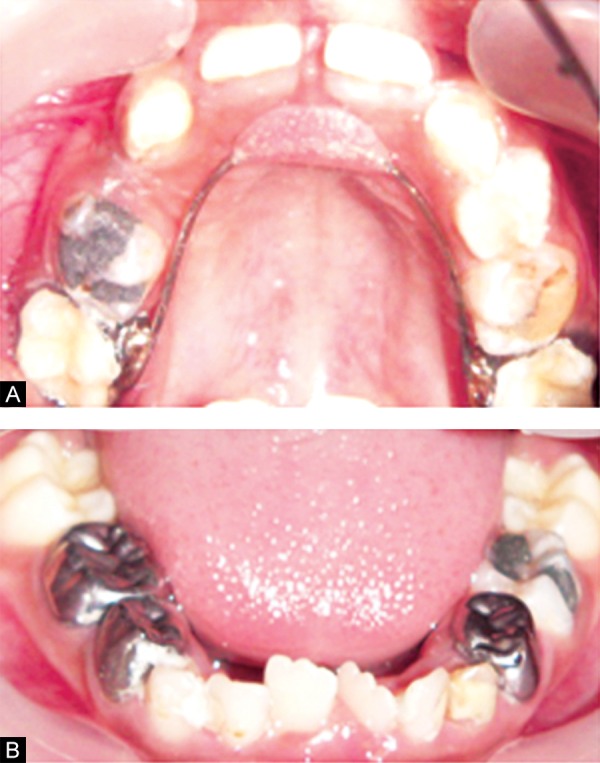
Postoperative view after 18 months follow-up (A) upper arch, (B) lower arch

Eighteen months follow-up showed complete eruption of permanent maxillary centrals (11, 21) with the correction of anterior crossbite. With the eruption of permanent maxillary first molar (16, 26) band and loop space maintainer was replaced by Nance palatal arch appliance (Figs 4A and B).

**Figs 5A and B:**
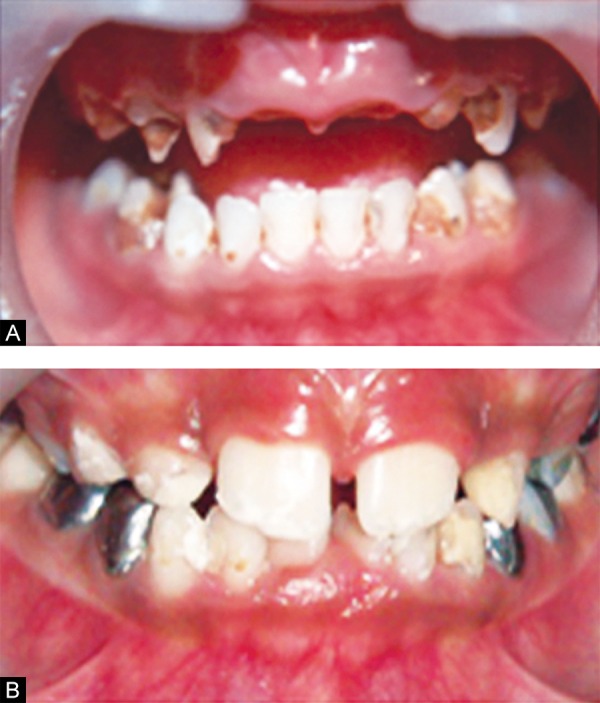
(A) Preoperative view (first visit), (B) postoperative view (follow-up)

Recall visits were planned every 3 months initially followed by biannual visits, during which we observed improvement in the patient’s oral health. Dietary and behavioral changes were satisfactory (Figs 5A and B). The oral rehabilitations, including preventive and curative measures were found to be successful in our patient at the end of 18 months follow-up period.

## DISCUSSION

Dental caries is one of the most common diseases affecting mankind. Almost every individual is susceptible to dental caries. However, caries is more prevalent in the younger population and considered a disease of childhood. Severeearly childhood caries describes dental caries in the primary dentition of young children that occur abruptly, spread widely and rapidly, and is burrowing in nature resulting in early involvement of the dental pulp.^6^ In young infants, this multifactorial disease is especially associated with the frequent use of sweetened fluids and fermentable carbohydrates over extended periods, poor oral hygiene as well as high level of mutans streptococci infection.^5^

As young children are usually anxious about dental treatment, their level of cooperation is limited, leading to a challenging situation.^5^ Anxiety or fearfulness is known to affect the child’s behavior, and largely determines the success of a dental appointment. Our patient who was reluctant and anxious in the 1st visit was counseled and modeled through behavior modification techniques, which paved the way towards future treatment.

S-ECC may be prevented by early counseling. The first step is to initiate treatment of all carious lesions to stop or at least slow the progression of the disease and to identify the most important causes of the existing condition.^7^ Intercessions before undesirable behaviors begin are preferable to attempt to alter deeply ingrained harmful habits.^8^ So, in the present case, the aim of first clinical intervention was to reduce caries activity. Therefore, detailed parental education, appropriate dietary counseling and changes in oral hygiene were initiated.

As our patient was on high cariogenic diet, they were advised to lower the frequency of carbohydrate ingestion rather than reducing the total carbohydrate intake. Although it is neither feasible nor desirable to eliminate sugar completely from the diet, we recommended that between meal snacks be supervised by parents and that, if possible, sugar intake be limited to meal times.^9^ They were also asked to include cheese in their diet as eating cheese will bathe the teeth in calcium, phosphate and bicarbonates, which in turn increase the pH of plaque and foster remineralization.^3,7,10^

Fluoride is highly effective in remineralizing enamel and inhibiting the progression of incipient lesions.^11^ The periodic professional topical applications of more concentrated fluoride solution, gels/varnishes have repeatedly demonstrated significant reduction in the incidence of dental caries in children as well as arrestment of incipient lesions.^7^ Fluoride varnish was chosen for topical application because it covers the teeth with an adherent film, enhancing the uptake of slowly released fluoride ions into the tooth structure and providing a protective effect against caries incidence.^8^

It must be emphasized to the parents that treating a carious tooth by providing a restoration will not cure the disease. If the unfavorable oral environment that caused the cavity persists, so the disease and more restorations will be required on time. So they must be instructed to follow strict preventive regimen.^4,12^

Restorative treatment should always be used in conjunction with preventive therapy, based on the child’s risk factors and age.^6^ Caries stabilization and provisional restorations should be in symptom-free teeth with established dentinal caries to minimize the risk of pulpal exposure in the future and to improve function.^13^ Gross caries excavation as an initial approach in the control of caries helps temporarily arrest caries process and prevents its rapid progression to the dental pulp.^7^ When cavitation has occurred, more definitive treatment is required. Depending on the extension of the lesion, pulpal therapy followed by stainless steel crowns/strip crowns or extractions may be indicated.^4,12^ After extraction, prosthesis should be provided for space maintenance, function and esthetics. In our case, extractions, pulpectomies and conventional glass ionomer restorations were performed to improve the oral health condition. In addition, space main-tainer and stainless steel crowns were also placed.^14^

Space maintainer was given to avoid space loss problems. Stainless steel crowns were chosen due to their ability to accurately resemble the anatomy of primary molars, to successfully restore occlusion and to provide adequate food grinding. Due to the patient’s high caries risk, the smoothness of the crown contributes to reduction in bacterial biofilm accumulation, favoring better oral hygiene.^5^

Most developing crossbites, when recognized at an early stage, have been successfully treated by tongue blade therapy which was instituted in our case.^15^ It is used to treat a tooth anterior crossbite. It works best with a central incisor that has only recently erupted into crossbite, because there is minimal overbite and minimal disturbance in the periodontium of the opposing lower central incisor, which is usually forced labially as the teeth close in their ‘locked bite’.^16^

Success of the treatment depends mainly on recall visits. The recall appointments should be set at each visit based on the clinician’s judgment of the patient’s risk for future disease at that time.^7^ Thus, the importance of follow-up visits should be reiterated to the parents.^17^

Treating caries at the level of the disease process to prevent its expression, progression and ultimate dental destruction is the highest calling and deepest challenge facing dentists who treat young children. Success in delaying caries initiation and suppressing its expression holds the greatest promise for children’s long-term oral health. A disease free mouth brings happiness and satisfaction not only to the parents and to children but also to the dental team who provided the information, instruction and reinforcement.^9,18^ Complete cooperation from the patient and parent contributed to the success of the treatment.

## CONCLUSION

S-ECC is a multifactorial disease that has numerous biological, psychosocial and behavioral risk factors. Children having caries in their primary teeth are more likely to develop caries in their permanent teeth. Although ECC can be arrested, early detection is of paramount importance. Thus, complete oral rehabilitation with effective preventive measures advocated during their childhood will help to reduce future risk for caries. Not only pediatric dentists but also the medical and the dental professionals as a whole should be united in the fight against this rapidly spreading condition.
